# Screening for Neuroprotective and Rapid Antidepressant-like Effects of 20 Essential Oils

**DOI:** 10.3390/biomedicines11051248

**Published:** 2023-04-23

**Authors:** Khoa Nguyen Tran, Nhi Phuc Khanh Nguyen, Ly Thi Huong Nguyen, Heung-Mook Shin, In-Jun Yang

**Affiliations:** Department of Physiology, College of Korean Medicine, Dongguk University, Gyeongju 38066, Republic of Korea; trannguyen053@dgu.ac.kr (K.N.T.); npkhanhnhi@dgu.ac.kr (N.P.K.N.); nguyenthihuongly_t58@hus.edu.vn (L.T.H.N.)

**Keywords:** depression, anxiety, rapid-acting effect, essential oil, glutamate, neurotoxicity, neuroinflammation

## Abstract

Depression is a serious psychiatric disorder with high prevalence, and the delayed onset of antidepressant effects remains a limitation in the treatment of depression. This study aimed to screen essential oils that have the potential for rapid-acting antidepressant development. PC12 and BV2 cells were used to identify essential oils with neuroprotective effects at doses of 0.1 and 1 µg/mL. The resulting candidates were treated intranasally (25 mg/kg) to ICR mice, followed by a tail suspension test (TST) and an elevated plus maze (EPM) after 30 min. In each effective essential oil, five main compounds were computationally analyzed, targeting glutamate receptor subunits. As a result, 19 essential oils significantly abolished corticosterone (CORT)-induced cell death and lactate dehydrogenase (LDH) leakage, and 13 reduced lipopolysaccharide (LPS)-induced tumor necrosis factor alpha (TNF-α) and interleukin 6 (IL-6). From in vivo experiments, six essential oils decreased the immobility time of mice in the TST, in which *Chrysanthemum morifolium* Ramat. and *Myristica fragrans* Houtt. also increased time and entries into the open arms of the EPM. Four compounds including atractylon, α-curcumene, α-farnesene, and selina-4(14),7(11)-dien-8-one had an affinity toward GluN1, GluN2B, and Glu2A receptor subunits surpassed that of the reference compound ketamine. Overall, *Atractylodes lancea* (Thunb.) DC and *Chrysanthemum morifolium* Ramat essential oils are worthy of further research for fast-acting antidepressants through interactions with glutamate receptors, and their main compounds (atractylon, α-curcumene, α-farnesene, and selina-4(14),7(11)-dien-8-one) are predicted to underlie the fast-acting effect.

## 1. Introduction

Depression is a complex psychiatric disorder, characterized by fatigue, constant feelings of sadness, and loss of pleasure [1]. Statistics show that depression remains a major burden in society, affecting approximately 280 million people worldwide [2]. Several pathological mechanisms of depression have been documented, including alterations in neurotransmitter systems, hypothalamus–pituitary–adrenal (HPA) axis activity, neuroinflammation, and changes in brain structures [3]. Norepinephrine and dopamine are neurotransmitters that play a role in reward processing and motivation, and their abnormal serum levels have been reported in depressed rodents [4]. Although serotonin has been the most extensively studied neurotransmitter in depression, researchers recently offer compelling data rejecting the relationship between serotonin activity to depression [5]. Stress-induced biological alterations, therefore, are receiving more attention as significant contributors to depression [6]. The HPA axis is a complex system that regulates the body’s stress response. Dysregulation of the HPA axis and the resulting elevations in corticotrophin-releasing hormone, adrenocorticotropic hormone, and cortisol or corticosterone (CORT) levels have been proposed as key mechanisms underlying the development of depression [7,8]. A dysregulated HPA axis activity is proposed to be accompanied by the overproduction of pro-inflammatory cytokines such as tumor necrosis factor alpha (TNF-α), interleukin (IL)-6, or IL-1β, which leads to the loss of hippocampal neurogenesis and promotion of depression-like behaviors [9,10]. In addition, chronic exposure to glucocorticoids such as cortisol or CORT can lead to dysfunction prefrontal cortex or atrophy of the hippocampus and structural deficits in the dentate gyrus area, which are all brain regions responsible for mood regulation [11,12]. In clinical practice, classical antidepressants exert their effects by inhibiting neurotransmitter-degrading enzymes (monoamine oxidase inhibitors) or inhibiting neurotransmitter reabsorption (selective serotonin reuptake inhibitors (SSRIs), selective serotonin and noradrenaline reuptake inhibitors, and tricyclic antidepressants) [13]. However, safety concerns have been raised, with side effects including weight gain, constipation, drowsiness, or even lethal hypertension [14,15]. Furthermore, the delayed onset of antidepressant action is another major limitation. For instance, acute treatment with SSRIs was found to initially elevate serotonin levels only in the cell body and dendrites, not in axons, which then immediately inhibit serotonin neuronal firing via an action at 5HT1A somatodendritic autoreceptors. In long-term treatment, SSRIs can cause a desensitization of 5HT1A autoreceptors, increasing the firing rate of neurons and serotonin release at axon terminals to postsynaptic receptors [16]. This partly explains the slow onset response of existing antidepressants, which usually take two to three weeks to manifest their effects [17]. Therefore, these limitations lead to an urgent need for safer agents with rapid antidepressant action.

In recent years, there has been a growing interest in research using essential oils due to their purported ability to alleviate a wide range of health issues, including inflammation, cancer, insomnia, anxiety, and depression with fewer side effects [18]. Essential oil is a mixture of secondary metabolites derived from plants [19]. Over 60 components have been identified in essential oils, with major compounds being benzenoids, phenylpropanoids, and terpenoids [20]. Their small, lipophilic components can rapidly and easily penetrate the blood–brain barrier (BBB) to access brain tissues, interacting with the thalamus, cerebral cortex, and limbic system, suggesting their potential use in rapidly reducing the symptoms of anxiety and depression [21,22]. For instance, lavender and citrus essential oil exerted anxiolytic-like and antidepressant-like effects in rodent models by restoring the decrease of monoamine neurotransmitter levels with downregulation of BDNF in serum or in the hippocampus [23,24,25].

Intranasal administration has been emphasized as a noninvasive method for the rapid management of neuropsychiatric disorders [26]. Intranasal agents directly stimulate the olfactory and trigeminal chemoreceptors, further enhancing the production of neurotransmitters and regulating the neuroendocrine system. In addition, mucosal epithelial pathways with blood vessel-dense nasal mucosa also contribute to rapid substance absorption and subsequent systemic effects, avoiding the hepatic first-pass effect and thus improving drug bioavailability [26,27]. Intranasal delivery of berberine, curcumin, and genipin using a thermosensitive hydrogel system has been reported to improve depressant-like activities in rodent models by enhancing monoamine neurotransmitter concentrations in the hippocampus and striatum. Additionally, at a lower dosage, these treatments exerted superior effects compared with intragastric or intraperitoneal routes [28,29,30].

Ketamine is a widely known medication that can rapidly alleviate the symptoms of depression through interacting with *N*-methyl-D-aspartate receptors (NMDARs), including GluN1 and GluN2B receptor subunits [31]. Notably, the GluN2B subunit is involved in many neurological disorders, and therefore significantly responsible for the biophysical and pharmacological properties of the NMDARs [32]. Ifenprodil, a GluN2B-selective NMDAR antagonist, was also found to exert a rapid-acting antidepression-like effect, compared with traditional medications that regulate the monoaminergic system [33]. In addition to NMDARs, the role of alpha-amino-3-hydroxy-5-methyl-4-isoxazole propionic acid receptors (AMPARs) including GluA1 and GluA2 subunits was also revealed in the rapid and long-lasting effects of antidepressants, for instance, ketamine and TAK-653 that potentiate AMPAR activity in maintaining synaptic plasticity [34,35,36]. Therefore, studies on the rapid action of new antidepressants should consider mechanisms via NMDARs and AMPARs.

Against this background, the present study was performed to identify and recommend essential oils that might have a rapid antidepression-like effect. We examined the in vitro neuroprotective and anti-neuroinflammatory effects of 20 essential oils to investigate their potential antidepression-like effects. Potentially antidepressant essential oils were then evaluated via intranasal administration in ICR mice using the tail suspension test (TST) and elevated plus maze test (EPM). Molecular docking was subsequently performed to predict the interactions between the major compounds in the effective essential oils and NMDAR and AMPAR subunits.

## 2. Materials and Methods

### 2.1. Preparation of Essential Oils

Twenty herbs were purchased from Omni Herb (Daegu, Republic of Korea), and each herbal material was immersed in distilled water (1:10 *w*/*w*) and hydrodistilled for 4 h using a steam distillation solvent extraction (SDE) apparatus, and the distilled oil was captured in *n*-hexane (Chemicals Duksan Corp., Ansan, Gyeonggi, Republic of Korea). The oil was dehydrated using a separating funnel, and *n*-hexane was evaporated. The essential oil was weighed (*Thuja orientalis* L. (0.28% *w*/*w*), *Acorus gramineus* Sol. (1.79%), *Foeniculum vulgare* Mill. (1.09%), *Magnolia biondii* Pamp. (0.55%), *Ligusticum striatum* DC. (0.46%), *Prunella vulgaris* L. (0.03%), *Chrysanthemum morifolium* Ramat. (0.28%), *Cinnamomum cassia* (Nees & T.Nees) J.Presl (0.75%), *Zingiber officinale* Roscoe (0.11%), *Santalum album* L. (0.39%), *Nardostachys jatamansi* (D. Don) DC. (1.38%), *Angelica acutiloba* (Siebold & Zucc.) Kitag. (0.20%), *Aucklandia lappa* DC. (0.21%), *Mentha arvensis* L. (yield 0.73%), *Perilla frutescens* (L.) Britton (0.39%), *Syzygium aromaticum* (L.) Merr. & L.M.Perry (6.75%), *Citrus reticulata* Blanco (0.41%), *Atractylodes lancea* (Thunb.) DC. (0.11%), *Agastache rugosa* (Fisch. & C.A.Mey.) Kuntze (0.20%), *Myristica fragrans* Houtt. (0.87%)) and stored at −20 °C until further use.

### 2.2. Animal Experiments and Treatments

Five-week-old male ICR outbred mice weighing 25–29 g were obtained from Koatech Lab Animal Inc. (Pyeongtaek, Gyeonggi, Republic of Korea) and acclimated for one week before the behavioral test. All mice had free access to water and a commercial pellet diet (5L79; PMI Nutrition, St. Louis, MO, USA). All experimental animal procedures were approved by the Institutional Animal Care and Use Committee of Dongguk University (IACUC-2021-15). The mice were randomly divided into the following groups: control group (CON), essential oil treatment groups (EO), and memantine group (MEM), which were offered vehicle (saline and Tween 80, 3% *v*/*v*), essential oil (25 mg/kg), and memantine (3 mg/kg), respectively. On the day of the experiment, all animals were habituated to the test room for two hours prior to receiving a 10 µL intranasal administration (CON and EO groups) or 200 µL intraperitoneal injection (MEM group). Thirty minutes after the treatment, a behavioral test was performed.

### 2.3. Tail Suspension Test (TST)

The effect of essential oils on depression-like behaviors in mice using the TST test was based on the fact that rodents subjected to the short-term, inescapable stress of being suspended by their tail will develop an immobile posture [37]. The tail of the mouse was attached to a bar using 15 cm adhesive tape, and the distance from the bar to the ground was fixed at 50 cm. All 6 min trials were recorded, and the total mobility time of each mouse was measured by manual scoring. The strong shaking of the body and movement of the four limbs akin to running were counted as mobility, whereas small movements that were confined to the front legs but without the involvement of the hind legs were not. The total mobility time was then subtracted from the 360 s of test time and was then stated as the immobility time. After each trial, the suspension box was wiped with 70% ethanol to remove unwanted odors.

### 2.4. Elevated Plus Maze Test (EPM)

The EPM test, which is based on the innate tendency of rodents to avoid elevated and open spaces and explore novel environments, was performed to assess the anxiety-like behaviors of mice after essential oil treatment [38]. The equipment consisted of four 29 cm × 5 cm arms elevated 40 cm above the ground, with two “closed arms” enclosed by 14 cm black walls. Each mouse was placed in the central area of the maze for a 5-min free exploration period. The arms were cleaned with 70% ethanol after every trial to remove unwanted odors, urine, and feces. The distance traveled, number of entries into, and time spent in the open arms were recorded and analyzed using Smart V3.0 software (Panlab Harvard Apparatus, Holliston, MA, USA).

### 2.5. Cell Culture and Treatments

PC12 cells (a rat adrenal medullary pheochromocytoma cell line) were cultured in Roswell Park Memorial Institute (RPMI) medium (Welgene Inc., Gyeongsan, Gyeongsangbuk-do, Republic of Korea) supplemented with 10% fetal bovine serum (FBS) (Merck KGaA, Darmstadt, Germany) and 1% penicillin-streptomycin (Thermo Fisher Scientific, Waltham, MA, USA) at 37 °C in a 5% CO_2_ humidified environment. PC12 cells were incubated with the SDE extract of each essential oil (0.1 or 1 μg/mL) for 1 h and then stimulated with corticosterone (CORT) (200 μM) for 24 h.

BV2 cells (an immortalized mouse microglial cell line) were cultured in Dulbecco’s Modified Eagle Medium (DMEM) supplemented with 10% FBS (Merck KGaA, Darmstadt, Germany) and 1% penicillin-streptomycin (Thermo Fisher Scientific, Waltham, MA, USA) at 37 °C in a 5% CO_2_ humidified environment. For the cell viability assay, BV2 cells were treated with the SDE extract of each essential oil (0.1 or 1 μg/mL). To test the anti-neuroinflammatory effects of the essential oils, BV2 cells were pretreated with non-toxic doses of each herb extract for 6 h and then stimulated with lipopolysaccharide (LPS) (1 μg/mL) for 18 h.

### 2.6. Water-Soluble Tetrazolium Salt Assay (WST)

The effects of the essential oils on the viability of PC12 and BV2 cells were examined using the EZ-Cytox assay kit (DoGenBio, Seoul, Republic of Korea). Cells were seeded at a density of 5 × 10^4^ cells/100 µL/well in 96-well plates and incubated at 37 °C and 5% CO_2_ for 24 h. After treating the cells and incubating for 24 h, 10 μL of the WST solution was added to each well, and the plates were incubated for 3–4 h. Absorbances were measured at a detection wavelength of 450 nm and a reference wavelength of 650 nm using a Tecan microplate reader (Männedorf, Switzerland).

### 2.7. Lactate Dehydrogenase Assay (LDH)

LDH released into the culture supernatant was measured using an EZ-LDH assay kit (DoGenBio, Seoul, Republic of Korea). PC12 cells were seeded and incubated under the same conditions as those used for the WST assay. After 24 h of treatment, the cell culture plates were centrifuged at 600× *g* for 5 min. Subsequently, 10 µL of supernatant from each well was transferred to a new 96-well plate and mixed with 100 μL of LDH reaction mixture for 30 min in darkness. LDH levels in the culture medium were determined by measuring the absorbance at 450 nm (reference wavelength, 650 nm) using a Tecan microplate reader (Männedorf, Switzerland).

### 2.8. Enzyme-Linked Immunosorbent Assay (ELISA)

The levels of inflammatory cytokines TNF-α and IL-6 secreted by BV2 cells in the culture media were measured to evaluate the anti-inflammatory activity of the essential oils. The cells were seeded at a density of 2 × 10^5^ cells/1 mL/well in 12-well plates and incubated at 37 °C and 5% CO_2_ for one day. 24 h after treatments, cell culture media were collected and centrifuged at 1500× *g* rpm for 10 min at 4 °C to remove particulates. The next steps were performed using ELISA kits (LABISKOMA, Seoul, Republic of Korea) following the manufacturer’s protocols. The absorbance at 450 nm was measured to detect cytokine levels, using a Tecan microplate reader (Männedorf, Switzerland).

### 2.9. Molecular Docking

The crystal structures of the GluN1 and GluN2B-NMDAR subunits and GluA2-AMPAR subunit (PDB ID:5H8Q, 5EWM, and 5ZG3, respectively) were downloaded from the Protein Data Bank (RCSB PDB) as previously described [39,40,41]. Then, all the heteroatoms and water molecules of the proteins were removed. Finally, Kollman charges were added to the proteins, and the macromolecules were exported into a PDBQT format for molecular docking. Information about the main compounds of essential oils was collected from the PubMed database and Traditional Chinese Medicine Systems Pharmacology Database and Analysis Platform (TCMSP). Only compounds with a molecular weight ≤ 500 Da and blood–brain barrier index ≥ 0.3 were selected. The 3D structures of the compounds were retrieved from the PubChem database. All ligands were then converted into PDBQT format using AutoDockTools version 1.5.6 (The Scripps Research Institute, San Diego, CA, USA).

Molecular docking of GluN1, GluN2B, and GluA2 was performed using AutoDock Vina, version 1.2.0 (The Scripps Research Institute, San Diego, CA, USA). According to the native ligand, the grid boxes that covered the active sites of GluN1, GluN2B, and GluA2 were defined using the following parameters: center_x = 14.66, center_y = −14.25, center_z = −25.74, size_x = 30, size_y = 30, and size_z = 30; center_x = 81.00, center_y = 5.90, center_z = −32.42, size_x = 30, size_y = 30, and size_z = 30; and center_x = 33.47, center_y = −55.89, center_z = 19.58, size_x = 30, size_y = 30, and size_z = 30, respectively. Before the docking study was performed, the docking parameters and algorithm were validated by re-docking the native ligand to the target receptor. The re-docked conformation was then superimposed onto the co-crystallized one using Discovery Studio Visualizer 2021 (Dassault Systèmes BIOVIA, San Diego, CA, USA) and the root mean square deviation (RMSD) was calculated. An RMSD lower than 2 Å suggested that the method could consistently predict the natural conformation of the ligand receptor [42,43].

In this study, ketamine, ifenprodil, and TAK-653, allosteric NMDAR antagonists and an allosteric AMPAR potentiator, respectively, were selected as reference compounds to check whether they strongly interacted with proteins inside selected binding pockets. Within the same grid boxes, herbal compounds with good protein affinity were suggested to exert similar effects as the reference compounds. Favorable conformations were selected based on the lowest binding energy. Finally, Discovery Studio Visualizer 2021 was used to visualize the molecular interactions between proteins and ligands.

### 2.10. Statistical Analysis

All experiments were performed with at least three independent experiments. GraphPad Prism 8.0 (GraphPad Software, San Diego, CA, USA) was used for statistical analysis. The results are presented as means ± standard deviations (SDs) followed by statistical significance (two-tailed unpaired Student’s *t*-test) defined as a *p*-value < 0.05.

## 3. Results

### 3.1. Effects of Essential Oils on CORT-Induced Neurotoxicity in PC12 Cells

**Figure 1:**
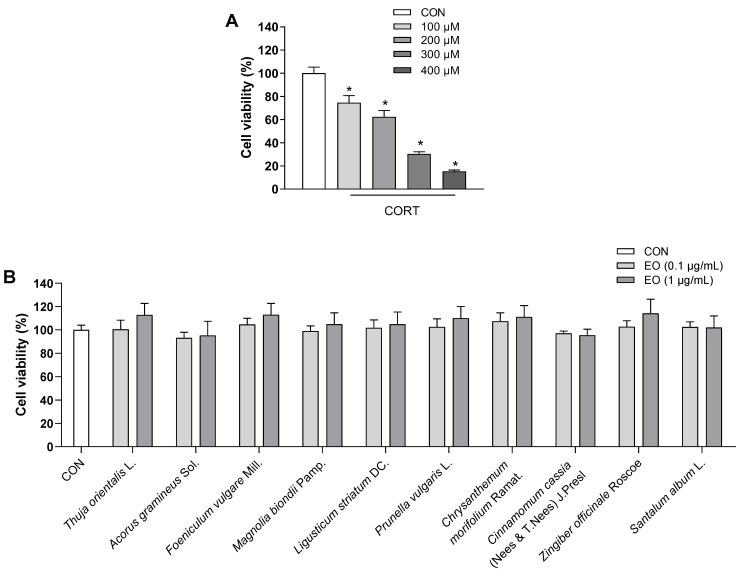

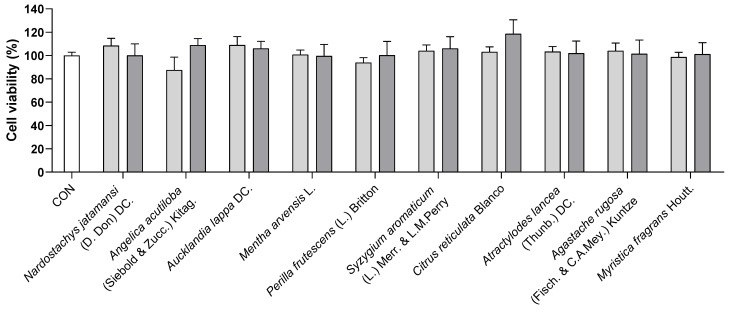
Effects of (**A**) CORT and (**B**) essential oils on the viability of PC12 cells. Results are presented as means ± SDs (*n* = 3 per experiment). * *p* < 0.05 vs. CON.

To determine the appropriate concentration of CORT for inducing cell damage, PC12 cells were incubated for 24 h with 100, 200, 300, and 400 µM CORT. Exposure to CORT reduced survival in a dose-dependent manner by 25, 38, 70, and 85%, respectively (Figure 1A). The cell viability of the 200 µM CORT-treated group decreased to about half that of the control group; this concentration was therefore used in subsequent experiments to induce neurotoxicity.

**Figure 2:**
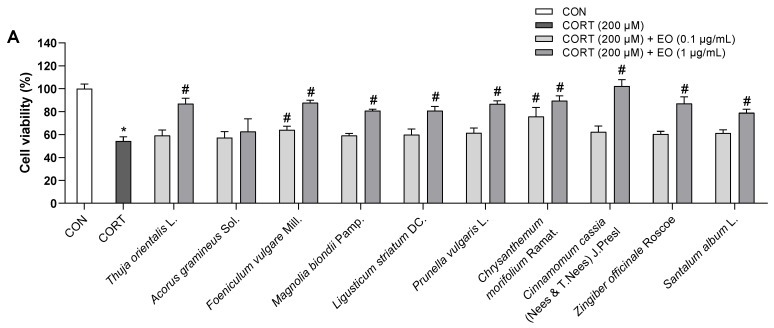

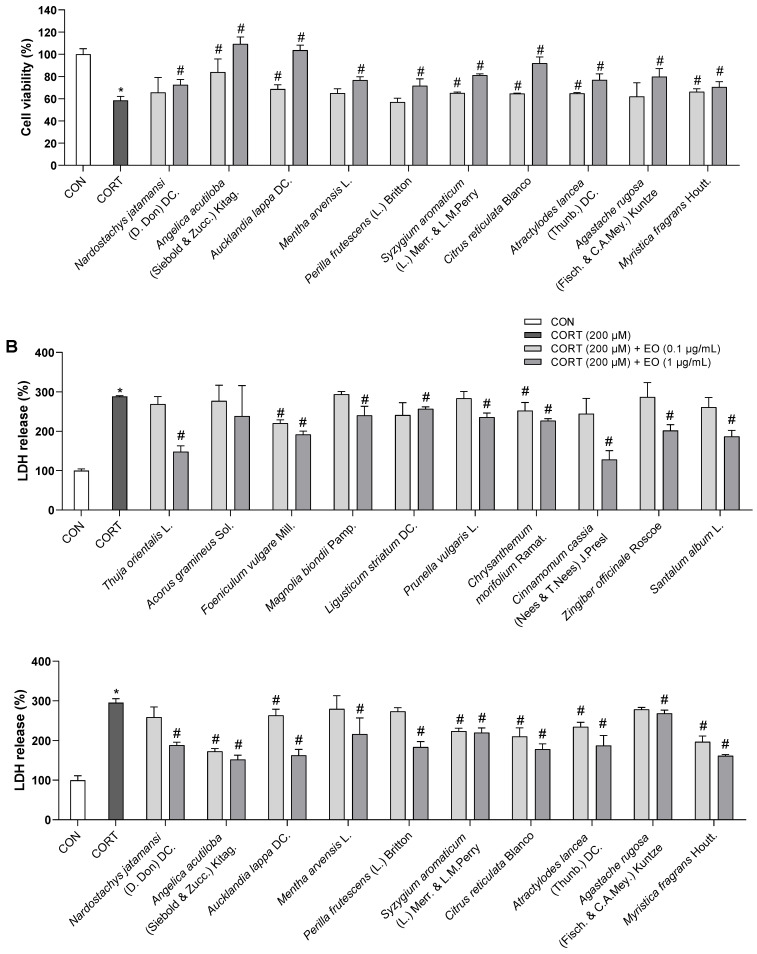
Effects of essential oils on CORT-induced neurotoxicity in PC12 cells. (**A**) Effects of essential oils on the cell viability in CORT-stimulated PC12 cells. (**B**) Effects of essential oils on the LDH release in CORT-stimulated PC12 cells. Results are presented as means ± SDs (*n* = 3 per experiment). * *p* < 0.05 vs. CON, # *p* < 0.05 vs. CORT-treated cells.

The effects of 0.1 and 1.0 µg/mL of each essential oil on the viability of PC12 cells were also assessed. As shown in Figure 1B, all essential oils did not cause toxicity to PC12 cells at doses of 0.1 and 1.0 µg/mL; these doses were then used to treat the cells for 1 h prior to incubation with CORT for 24 h. As shown in Figure 2A, compared with the control group, the cell survival rate significantly decreased in the model group treated with 200 µM CORT (*p* < 0.05). *Foeniculum vulgare* Mill., *Chrysanthemum morifolium* Ramat., *Angelica acutiloba* (Siebold & Zucc.) Kitag., *Aucklandia lappa* DC., *Syzygium aromaticum* (L.) Merr. & L.M. Perry, *Citrus reticulata* Blanco, *Atractylodes lancea* (Thunb.) DC., and *Myristica fragrans* Houtt. essential oils showed a dose-response relationship, while *Acorus gramineus* Sol. did not exhibit a protective effect at either of the two doses, and the remaining 11 essential oils exerted an effect only at the higher dose.

As LDH leakage from cells is widely used as a cellular damage marker, LDH release was measured in the culture medium to assess PC12 cell injury. LDH secreted from cells was significantly increased in the model group compared to that in the control group; the percentage of LDH leakage increased from 100% (control) to 288 ± 6.9%. However, pretreatment with essential oils reduced LDH leakage (Figure 2B), which was consistent with the data from the WST assay. These results indicated that essential oil pretreatment could prevent CORT-induced injury in PC12 cells.

### 3.2. Effects of Essential Oils on LPS-Induced Neuroinflammation in BV2 Cells

**Figure 3:**
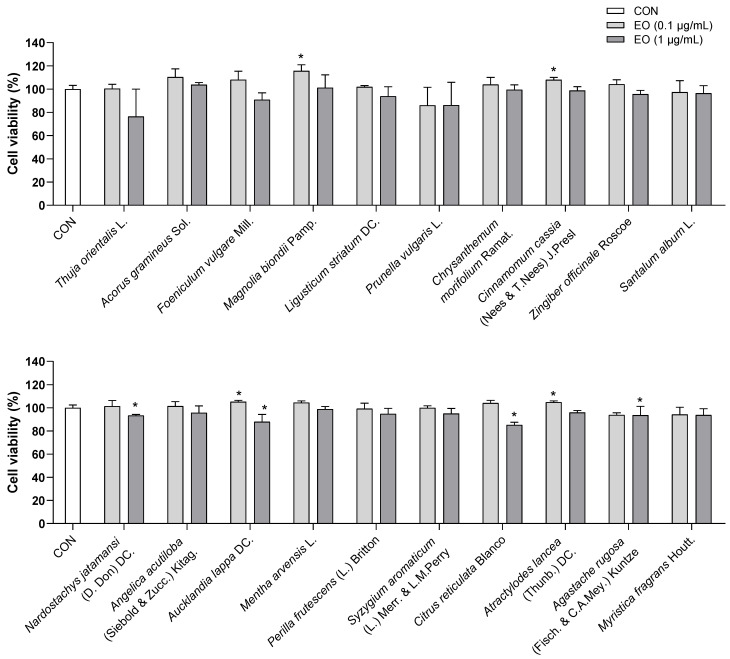
Effects of essential oils on the viability of BV2 cells. Results are presented as means ± SDs (*n* = 3 per experiment). * *p* < 0.05 vs. CON.

To test whether essential oil treatments were toxic to the viability of BV2 cells, a WST assay was performed using concentrations of 0.1 and 1.0 µg/mL of each essential oil. From the results, all essential oils did not cause toxicity to BV2 cells at doses of 0.1 and 1.0 µg/mL. Although *Thuja orientalis* L. at 1.0 µg/mL decreased cell viability to approximately 75%, this reduction was not statistically significant (Figure 3). Hence, doses of 0.1 and 1.0 µg/mL of all essential oils were used for the subsequent experiments.

**Figure 4:**
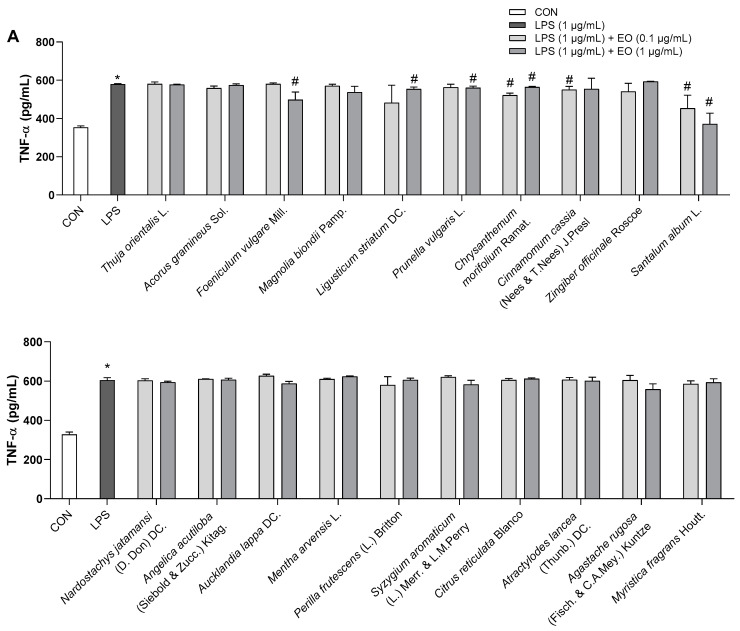

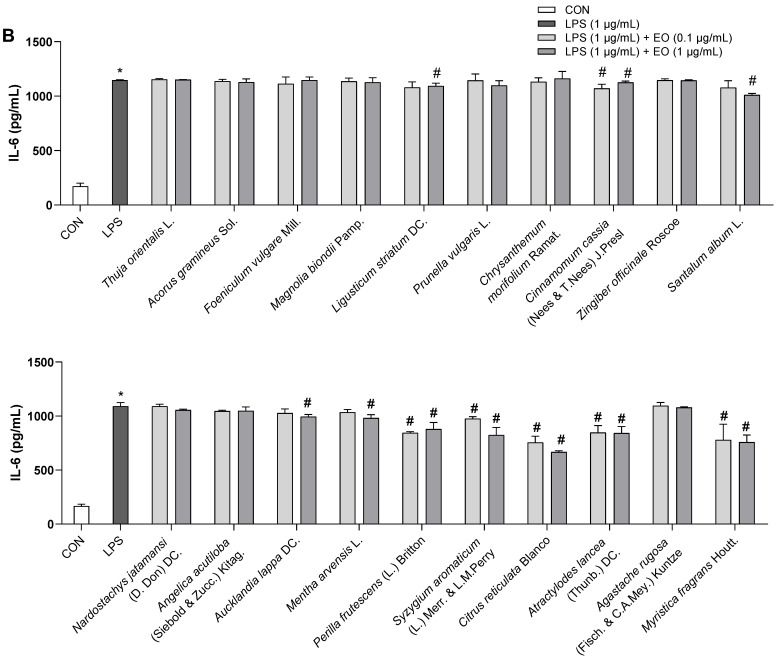
Effects of essential oils on LPS-induced neuroinflammation in BV2 cells. (**A**) TNF-α levels (pg/mL). (**B**) IL-6 levels (pg/mL). Results are presented as means ± SDs (*n* = 3 per experiment). * *p* < 0.05 vs. CON, # *p* < 0.05 vs. LPS-treated cells.

As shown in Figure 4, LPS (1 µg/mL) remarkably increased the production of the inflammatory cytokines TNF-α and IL-6 compared to that in the control BV2 cells, but this effect was blunted by pretreatment with essential oils at different levels. In particular, 10 essential oils (*Ligusticum striatum* DC., *Cinnamomum cassia* (Nees & T.Nees) J.Presl, *Santalum album* L., *Aucklandia lappa* DC., *Mentha arvensis* L., *Perilla frutescens* (L.) Britton, *Syzygium aromaticum* (L.) Merr. & L.M. Perry, *Citrus reticulata* Blanco, *Atractylodes lancea* (Thunb.) DC., and *Myristica fragrans* Houtt.) reduced IL-6 release, yet only six essential oils (*Foeniculum vulgare* Mill., *Ligusticum striatum* DC., *Prunella vulgaris* L., *Chrysanthemum morifolium* Ramat., *Cinnamomum cassia* (Nees & T.Nees) J.Presl, and *Santalum album* L.) abrogated the increase in TNF-α levels (Figure 4). Of the 20 examined essential oils, eight including *Chrysanthemum morifolium* Ramat., *Cinnamomum cassia* (Nees & T.Nees) J.Presl, *Santalum album* L., *Perilla frutescens* (L.) Britton, *Syzygium aromaticum* (L.) Merr. & L.M. Perry, *Citrus reticulata* Blanco, *Atractylodes lancea* (Thunb.) DC., and *Myristica fragrans* Houtt. suppressed TNF-α or IL-6 production at a low dose of 0.1 µg/mL. Moreover, three essential oils (*Ligusticum striatum* DC., *Cinnamomum cassia* (Nees & T.Nees) J.Presl, and *Santalum album* L.) significantly decreased the levels of both the assessed inflammatory cytokines (Figure 4). At the investigated doses, no beneficial effects were observed for *Thuja orientalis* L., *Acorus gramineus* Sol., *Magnolia biondii* Pamp., *Zingiber officinale* Roscoe, *Nardostachys jatamansi* (D. Don) DC., *Angelica acutiloba* (Siebold & Zucc.) Kitag., and *Agastache rugosa* (Fisch. & C.A.Mey.) Kuntze.

### 3.3. Rapid-Acting Effect of Essential Oils on Behavior Changes of Mice in the TST and EPM

**Figure 5:**
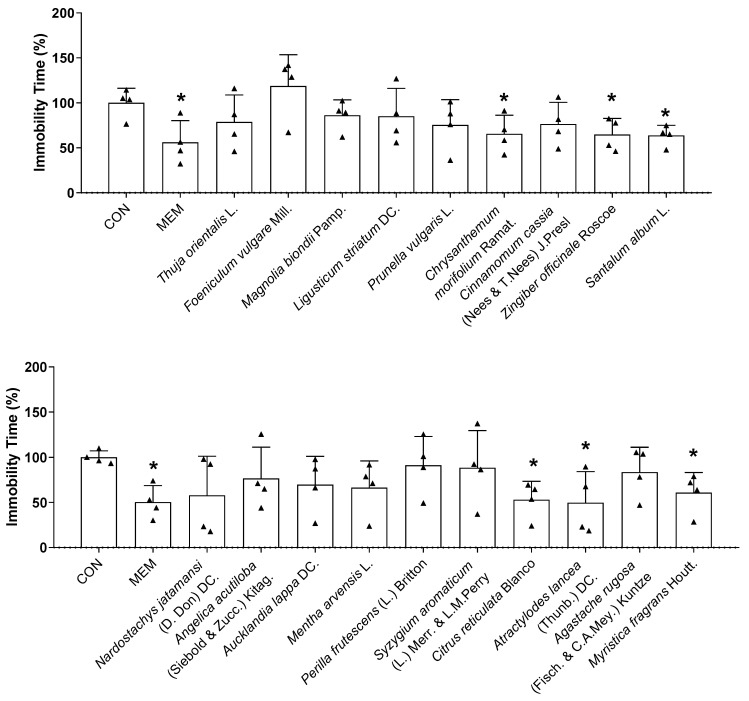
Effects of essential oils on the behavioral changes of mice in TST test. The immobility time in TST was recorded. Each black triangle indicates the value of each mouse subject in groups. Results are presented as means percentage relative to control ± SDs (*n* = 4 mice per group). * *p* < 0.05 vs. CON.

**Figure 6:**
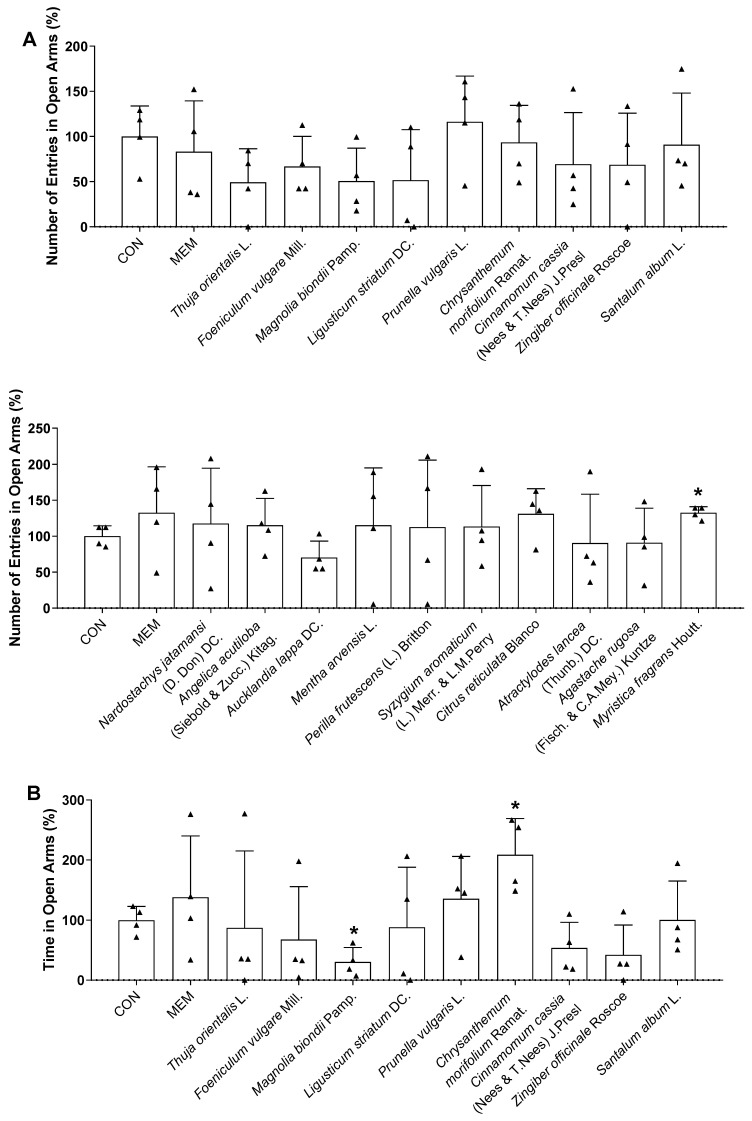

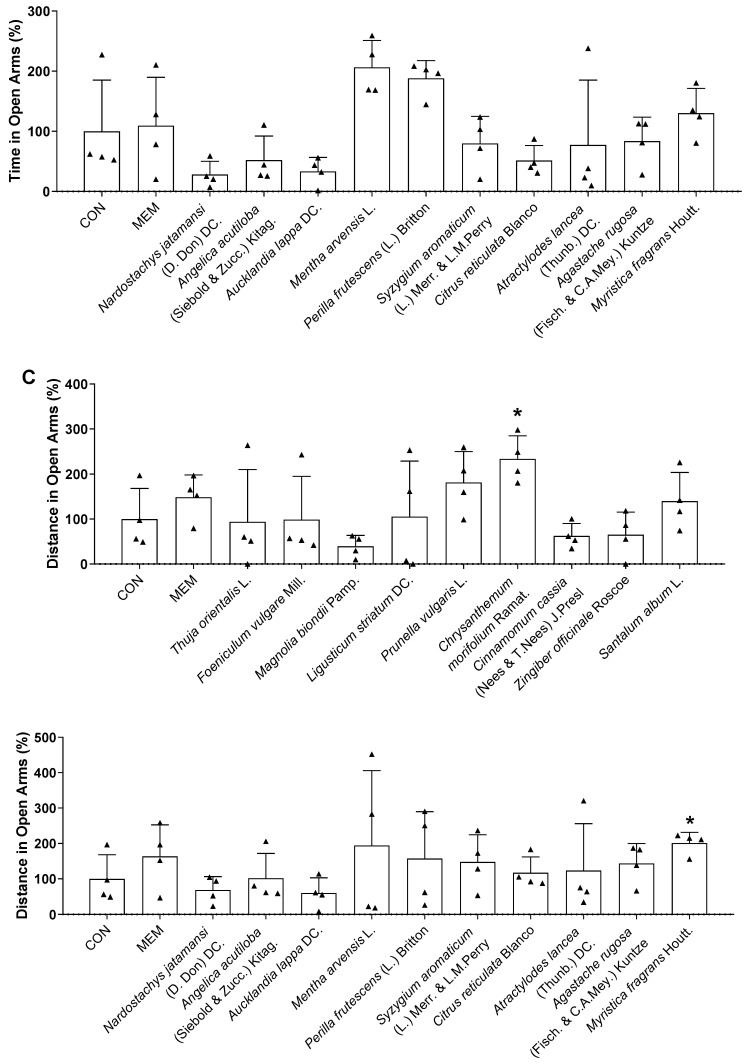
Effects of essential oils on the behavioral changes of mice in EPM test. The (**A**) number of entries, (**B**) time, and (**C**) distance in open arms were recorded. Each black triangle indicates the value of each mouse subject in groups. Results are presented as means percentage relative to control ± SDs (*n* = 4 mice per group). * *p* < 0.05 vs. CON.

Based on the in vitro results, 19 potential essential oils were used for the in vivo experiments. Thirty minutes after intranasal administration, the TST and EPM were conducted to investigate the effects of essential oils on the behavior of mice. During the TST, *Chrysanthemum morifolium* Ramat., *Zingiber officinale* Roscoe, *Santalum album* L., *Citrus reticulata* Blanco, *Atractylodes lancea* (Thunb.) DC., and *Myristica fragrans* Houtt. essential oil treatments exhibited antidepressant-like effects by decreasing the immobility time compared with the control mice (Figure 5). There was a reduction in the immobility time of mice in the TST after memantine treatment, while no statistically significant change was observed in the EPM (Figure 5 and Figure 6). As shown in Figure 6, both *Chrysanthemum morifolium* Ramat. and *Myristica fragrans* Houtt. essential oils enhanced the percentage of distance covered in the open arms in the EPM test. In addition, *Myristica fragrans* Houtt. significantly increased the number of open arm entries, while *Chrysanthemum morifolium* Ramat. treatment increased the time of mice exploring the open arms, indicating antianxiety-like effects. In contrast, *Magnolia biondii* Pamp. exerted the opposite effect, significantly reducing exploration time in the open arms.

### 3.4. Molecular Docking Analysis of Essential Oil Main Compounds

**Figure 7:**
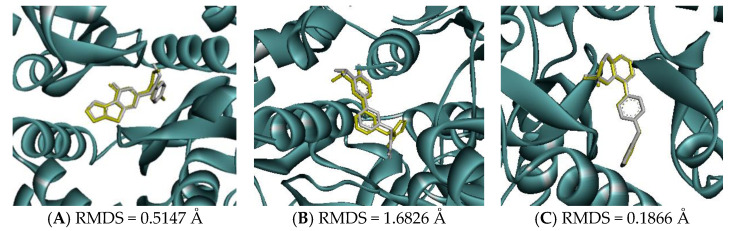
Superimposed zoomed-in image of native ligand and redocked native ligand in the active site of (**A**) GluN1, (**B**) GluN2B, and (**C**) GluA2 (native ligand: gray color, redocked ligand: yellow color).

**Figure 8:**
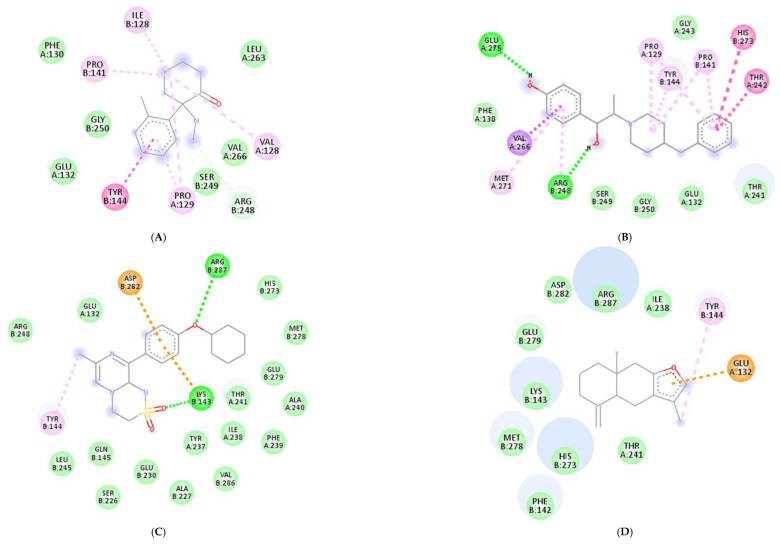

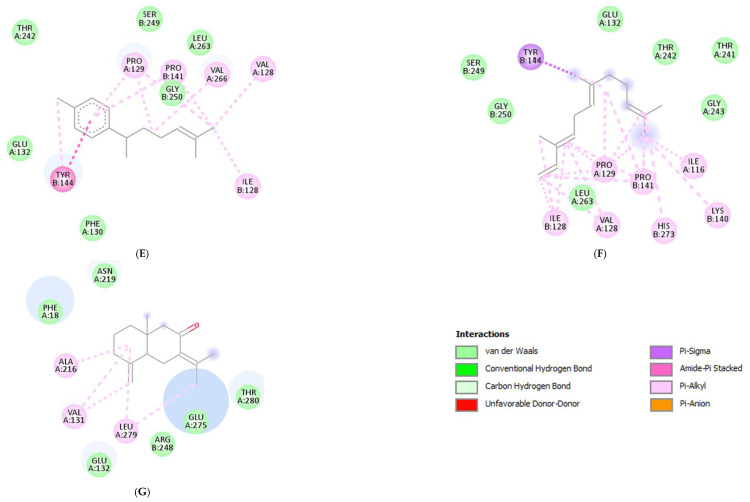
2D interactions between (**A**) ketamine, (**B**) ifenprodil, (**C**) TAK-653, (**D**) atractylon, (**E**) α-curcumene, (**F**) α-farnesene, and (**G**) selina-4(14),7(11)-dien-8-one with GluN1 protein.

**Figure 9:**
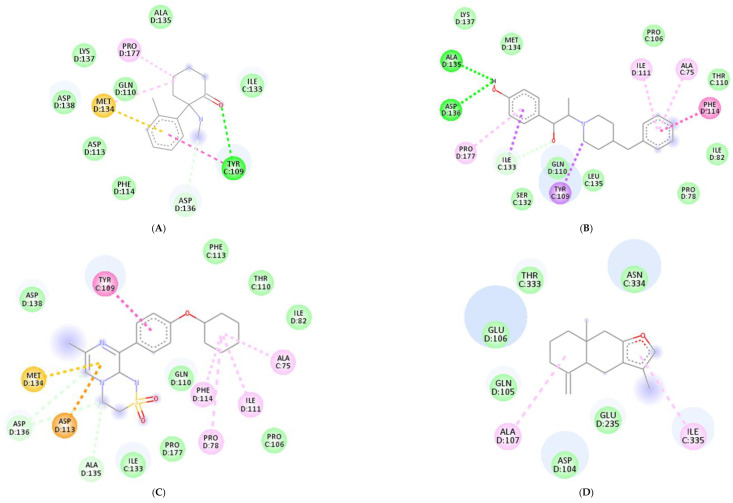

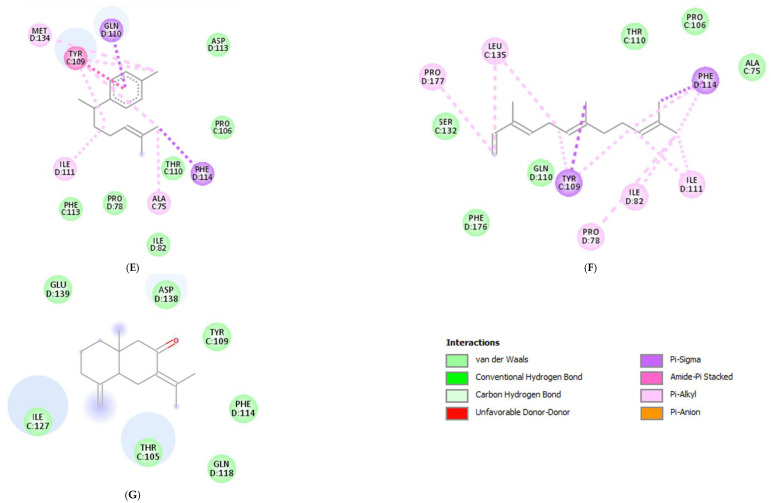
2D interactions between (**A**) ketamine, (**B**) ifenprodil, (**C**) TAK-653, (**D**) atractylon, (**E**) α-curcumene, (**F**) α-farnesene, and (**G**) selina-4(14),7(11)-dien-8-one with GluN2B protein.

**Figure 10:**
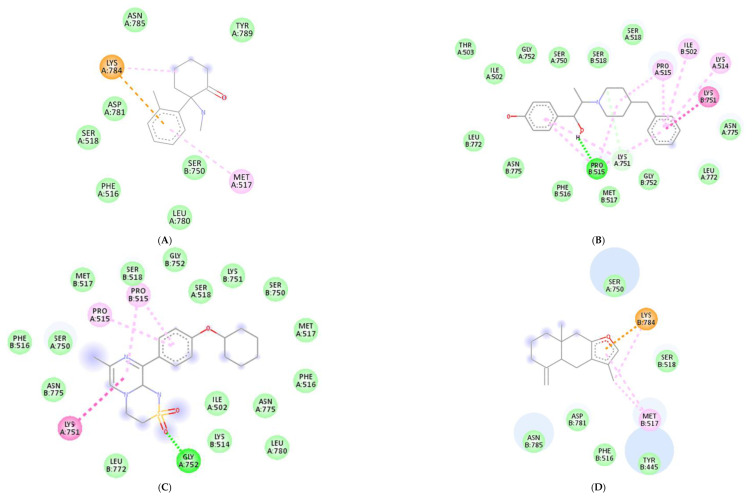

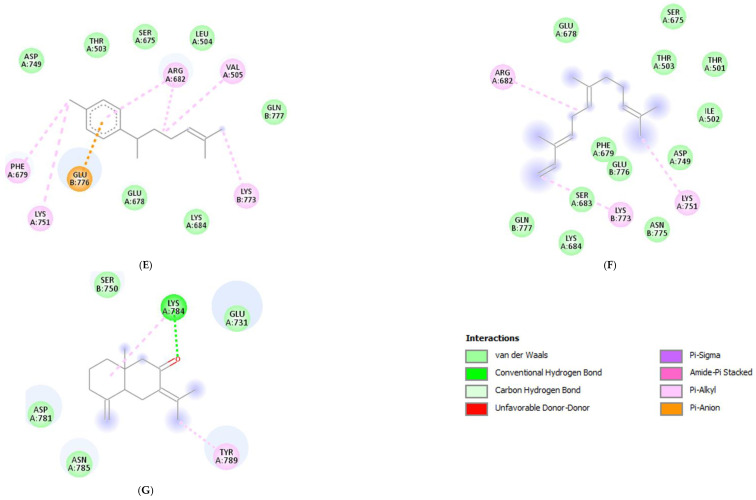
2D interactions between (**A**) ketamine, (**B**) ifenprodil, (**C**) TAK-653, (**D**) atractylon, (**E**) α-curcumene, (**F**) α-farnesene, and (**G**) selina-4(14),7(11)-dien-8-one with GluA2 protein.

Molecular docking was conducted to investigate the five major compounds of the seven essential oils that modified depressant- or anxiety-related behaviors in animal tests (Table 1). After removing duplicates, 27 compounds remained (Table 2). The docking protocol was validated through a re-docking experiment using the native ligand. Root mean square deviation (RMSD) values of less than 2 Å were observed, suggesting that the ligand-receptor conformations had a high docking accuracy (Figure 7). The molecular docking of compounds against the GluN1, GluN2B, and GluA2 proteins obtained using Autodock Vina are presented in Table 3, with higher negative binding energies indicating better affinity between components. Among the 27 natural compounds, 24, 22, and 13 had docking scores of less than −6.0 kcal/mol toward GluN1, GluN2B, and GluA2, respectively, suggesting a highly stable complex. When compared with the reference compounds, four compounds, including atractylon, α-curcumene, α-farnesene, and selina-4(14),7(11)-dien-8-one, had lower energy binding than ketamine when interacting with all three proteins, while none had lower docking scores than ifenprodil and TAK-653. The 2D conformations are visualized in Figure 8, Figure 9 and Figure 10.

## 4. Discussion

Side effects and delayed onset of action are the limitations of current antidepressants. Ketamine was reported to alleviate depressive symptoms within hours post-treatment, but rapid-acting antidepressants have not yet made any other striking progress [65,66]. Essential oils possessing antianxiety and antidepressant properties with fewer side effects have been reported [67]. They mainly consist of small and lipophilic molecules, and therefore can quickly and easily bypass the BBB to target brain tissues, acting on the cerebral cortex, thalamus, and limbic system, indicating their potential application in alleviating symptoms of anxiety and depression in a rapid manner [21,22]. Additionally, recent studies have revealed the advantages of intranasal administration over other routes of administration [26]. Hence, the purpose of this study was to identify essential oils with rapid-acting antidepressant effects when administered intranasally. As expected, our results suggested that different essential oils exerted neuroprotective effects in vitro and rapid antidepressant-like effects in a normal mouse model (Table 4). In addition, the effects of essential oils were comparable to those of MEM, a common medication used to treat moderate-to-severe dementia, whose acute antidepressant-like effects have also been reported in previous studies [68,69].

Neuroinflammation is a common feature in patients with depression. Several studies have reported that inflammation in the brain and the associated over-release of pro-inflammatory cytokines TNF-α, IL-6, or IL-1β can lead to a loss of hippocampal neurogenesis, promoting depression-like behaviors [10]. The underlying mechanism may be related to microglial cells, which influence neuronal excitability and neurotransmission [70]. Under threats such as the LPS challenge, microglia are activated to undergo morphological changes and secrete pro-inflammatory cytokines [71]. These cytokines can decrease the levels of serotonin and norepinephrine, which are neurotransmitters involved in mood regulation [72]. In addition, pro-inflammatory cytokines can affect the function of the hippocampus, a brain region that is important for learning, memory, and mood regulation [12]. In the present study, we treated the murine microglial cell line BV2 with LPS and essential oils, with the hypothesis that essential oils that could reduce LPS-induced pro-inflammatory cytokines would have potential antidepressant effects. Essential oils such as *Chrysanthemum morifolium* Ramat., *Santalum album* L., *Citrus reticulata* Blanco, and *Atractylodes lancea* (Thunb.) DC., which inhibited TNF-α or IL-6 released from LPS-treated BV2 cells, significantly decreased the immobility time of mice in TST, suggesting their antidepressant effects occurred through inactivating glial cells and reducing neuroinflammation.

Depression is also accompanied by the overproduction of CORT, which affects numerous physiological processes in neurons and astrocytes, such as apoptosis and synaptic plasticity [73]. High concentrations of CORT can increase the production of reactive oxygen species (ROS) and pro-inflammatory cytokines, which can damage neuronal cells [74,75]. Additionally, through activating glucocorticoid receptors (GRs) in neurons, CORT can cause a cascade of apoptosis-related enzymes known as caspases that are responsible for initiating programmed cell death [76]. GRs are mainly distributed in the hippocampus, and CORT-induced GR overexpression can lead to structural changes such as hippocampal atrophy, loss of dendritic synapses, and synaptic plasticity alternation, resulting in the development of anxiety and depression [77,78,79,80,81]. In this study, PC12 cells, a classical neuronal cell line, were treated with CORT to mimic the features of depression, and essential oils that protect cells from CORT-induced toxicity may have the potential to treat depression. PC12 cell viability was evaluated using a WST assay based on mitochondrial succinate dehydrogenase activity, indicative of cellular energy capacity, compared to LDH leakage, indicative of apoptosis [82]. Our results showed that 19 of the 20 essential oils included in this study inhibited CORT-induced cell death and LDH leakage, indicating their potential in the treatment of depression, probably by decreasing neuronal apoptosis. In addition, a very small dose of essential oil (SDE extract), including 0.1 or 1 µg/mL, was sufficient to exhibit anti-inflammatory and neuroprotective effects. Previous studies using ethanol or methanol extracts of herbal materials normally required an effective dose of 10–100 µg/mL. This demonstrates one benefit of essential oils in drug development applications [83,84,85,86].

The TST is an important behavioral model that is widely used to screen for new antidepressants. In animals subjected to inescapable stress, immobility has been suggested as a key indicator of behavioral despair, reminiscent of depressive disorders in humans [37]. In the present study, a significant decrease in immobility time in treated mice was observed with six essential oils only 30 min after intranasal administration, indicating their fast antidepressant-like activity. This is the first time the rapid antidepressant effects of *Chrysanthemum morifolium* Ramat., *Zingiber officinale* Roscoe, *Santalum album* L., *Citrus reticulata* Blanco, *Atractylodes lancea* (Thunb.) DC., and *Myristica fragrans* Houtt. essential oils have been reported, particularly through intranasal administration. Although the antidepressant activity of nutmeg (*Myristica fragrans*) seeds has been reported in previous studies, its effect was observed after oral administration for three days [87,88].

The EPM is an extensively used model for the assessment of novel anxiolytic agents, based on the natural tendency of rodents to avoid open and elevated areas [38]. Because high levels of anxiety result in depression, and that approximately 85% of patients with depression have significant anxiety, anxiety is considered one of the symptoms of depression [89,90]. Among the two effective essential oils, *Chrysanthemum morifolium* Ramat. increased the time mice spent in the open arms, whereas *Myristica fragrans* Houtt. increased open-arm entries. Although no essential oils significantly elevated either parameter, increasing trends were observed. Moreover, mice treated with the two essential oils also showed notably higher total distances in the open arms than that demonstrated in the control group. Therefore, these essential oils might have exerted anti-anxiety effects to a certain extent, as evidenced by an increased exploration in the open elevated space, which contributed to the reduction of depression-like behaviors.

Ketamine and ifenprodil, an NMDAR antagonist and a GluN2B-selective NMDAR antagonist, respectively, have been proven to be rapid-acting antidepressant therapies in clinical and in vivo studies. Their mechanisms may be related to the de-inhibition of mTOR activity, which induces protein biosynthesis, leading to synaptic potentiation [33,91]. In addition, by blocking spontaneous NMDAR-mediated neurotransmission, ketamine can suppress eukaryotic elongation factor 2 (eEF2) kinase function, preventing eEF2 substrate phosphorylation and enhancing BDNF translation. Ketamine can also upregulate AMPAR, especially the GluA1 and GluA2 subunits, thereby enhancing synaptic strengthening and transmission [91,92]. TAK-653, an AMPAR potentiator, exerts antidepressant effects in vivo. It can also directly activate AMPAR, stimulating the mammalian target of rapamycin (mTOR) and BDNF signaling in vitro [36]. In 2015, a study revealed that ketamine conferred fast antidepressant effects in mice 30 min after intraperitoneal injection, favorably suppressing NMDAR subunits GluN1 and GluN2B functioning in the hippocampus [31]. Additionally, Li et al. suggested that ketamine exerts antidepressant effects by enhancing AMPAR expression [34]. Indeed, current concepts regarding glutamate receptors suggest that drugs that increase AMPAR signaling or decrease NMDAR function may be effective antidepressants. Therefore, we performed a molecular docking analysis of the main compounds from the essential oils toward GluN1, GluN2B, and GluA2 proteins using ketamine, ifenprodil, and TAK-653 as reference compounds.

Molecular docking is a computational technique that predicts the binding orientation and affinity of small molecules (compounds) to a target protein or macromolecule (receptor subunits). It provides insights into the mechanisms of drug action and allows researchers to identify potential drug candidates and optimize their binding properties before conducting costly and time-consuming experiments [93,94]. Besides, through simulating the binding interactions between molecules and targets, the technique may also suggest whether a candidate drug has similar actions to other reference compounds. Here, we showed that in comparison with ketamine (with docking scores of −8.2, −5.9, and −4.9 kcal/mol toward GluN1, GluN2B, and GluA2, respectively), four compounds including atractylon, α-curcumene, α-farnesene, and selina-4(14),7(11)-dien-8-one had much lower docking scores, indicating stronger binding to the targeted receptors. Hence, these compounds that are main components of *Atractylodes lancea* (Thunb.) DC. and *Chrysanthemum morifolium* Ramat. essential oils are of greater concern.

*Atractylodes lancea* (Thunb.) DC. is a type of Atractylodis Rhizoma, whose extract has been commonly used for the treatment of digestive disorders owing to its anti-inflammatory and gastroprotective effects [59]. *Atractylodes lancea* (Thunb.) DC. may also have the potential to treat depression. A previous study suggested that drugs inhibiting gastric acid secretion can be used to treat depressive symptoms and antidepressants can be an effective treatment for stress ulcers, indicating a similar mechanism of pathogenesis shared by the two ailments [95]. This is consistent with our results showing that *Atractylodes lancea* (Thunb.) DC. essential oil significantly reduced depression-like behaviors in mice in the TST. Furthermore, atractylon, one of the major constituents of this essential oil, was recently found to improve cognitive dysfunction in mice by inhibiting microglial activation and exerting anti-inflammatory activity in vitro through the inhibition of cyclooxygenase-2 (COX-2) and inducible nitric oxide synthase (iNOS) expression [96,97]. Such an action has been previously revealed to curb depressive symptoms by reducing neuroinflammation [12,72]. Compared with ketamine, atractylon has a lower binding energy for the three glutamate receptor subunits, indicating a stronger binding capacity than ketamine. Notably, atractylon can interact with GluN1 and GluA2 subunits through the same amino acids as that of ketamine, suggesting a similar mechanism of action for its rapid-acting antidepressant effect (Figure 8 and Figure 10). Regarding selina-4(14),7(11)-dien-8-one, also a major component of *Atractylodes lancea* (Thunb.) DC., the pharmacological activity of this compound has not been reported to date. However, based on the molecular docking results, this compound was able to interact with the GluA2 subunit at sites similar to those of ketamine and ifenprodil (Figure 10). Hence, selina-4(14),7(11)-dien-8-one is also a compound with potential for the research and development of antidepressants.

*Chrysanthemum morifolium* Ramat. is a traditional herb widely used for the treatment of fever, headache, sore throat, and hypertension, due to the variety of flavonoids, anthocyanins, alkaloids, and phenolic acid components found in its extract [98]. Notably, neuroprotective and antioxidant effects, as well as its ability to reduce ROS levels and lipid peroxidation, have also been reported [99,100]. ROS and oxidative stress are known to cause dysfunction in neurotransmission and the HPA axis, reduced neuroplasticity, and neuroinflammation, all of which are involved in the pathogenesis of depression [101]. Hence, the antidepressant potential of *Chrysanthemum morifolium* Ramat. should not be overlooked. However, until now, only one previous study revealed the chronic antidepressant effect of *Chrysanthemum morifolium* on CORT-injected C57BL/6 mice, evidenced by significantly elevated sucrose consumption and serum serotonin levels [102]. Our results showed that *Chrysanthemum morifolium* Ramat. essential oil improved both anxiety-like and depression-like symptoms after only a single dose, suggesting an acute effect on depression. We predict that such an effect may originate from the two main compounds of the essential oil, α-curcumene, and α-farnesene, although only the anti-inflammatory effect of α-farnesene has been reported to date [103]. We found that the interaction of these two compounds with the glutamate receptor subunits were comparable to that of reference compounds. α-Curcumene and α-farnesene have binding energies for the three subunits surpassing that of ketamine, interact with GluN1 and GluN2B at many amino acids, similar to ketamine and ifenprodil, and form more bonds with the proteins than ketamine. This suggests that α-curcumene and α-farnesene not only bind well to NMDAR subunits but can also mimic the mechanisms by which ketamine and ifenprodil improve depression.

*Myristica fragrans* Houtt. or nutmeg is a well-characterized herb with various pharmacological properties such as antidiarrheal, antimicrobial, antifungal, antioxidant, cardioprotective, especially sedative and antidepressant effects [104]. However, to our best knowledge, this study is the first to report the neuroprotective effect in vitro as well as the rapid-acting antianxiety-like and antidepression-like effects in vivo of *Myristica fragrans* Houtt. essential oil via intranasal delivery. Unlike *Atractylodes lancea* (Thunb.) DC. and *Chrysanthemum morifolium* Ramat., fast actions of *Myristica fragrans* Houtt. appeared not to mainly exert via glutamate receptors since its main compounds such as limonene, β-myrcene, α-pinene, and β-pinene do not have a strong affinity towards GluN1 and GluA2 subunits. It should be noticed that not only glutamate receptors drive the rapid anxiolytic and antidepressant effects, but others might also do that function, for instance, gamma-aminobutyric acid B, or 5-hydroxytryptamine receptor 4 receptors [105,106,107]. A previous study showed that 3-day treatments of *Myristica fragrans n*-hexane extract reduced depression-like behaviors in mice by targeting the serotonergic and noradrenergic nervous systems [87,88]. Short-term inhalation of limonene has been found to restore chronic unpredictable mild stress-induced depression-like behavior by modulating the activity of the HPA axis, BDNF receptors, and monoamine neurotransmitter levels [23]. The acute anxiolytic effect of inhaled α-pinene after one day of treatment has also been reported, yet its underlying mechanism remains to be studied [108]. In short, though *Myristica fragrans* Houtt. essential oil is a potential material for rapid-acting antidepressant development, other mechanisms of action rather than glutamate pathway should be considered.

Notably, the rapid antidepressant-like effect of essential oils via intranasal administration is a novelty of this study. Nasal delivery has recently been shown to have advantages over other routes, such as oral administration or intraperitoneal injection. Since intranasal intervention directly transports exogenous materials from the nasal cavity to the brain, avoiding first-pass metabolism in the liver, it results in a fast onset of action and higher bioavailability of drugs [109,110]. In our study, intranasal administration of essential oils only 30 min prior to the behavioral test produced positive changes in the behavior of mice. We postulate that the olfactory bulb receives sensory information from the olfactory receptors in the lining of the nasal cavity and projects signals to certain brain regions, including the hippocampus and amygdala, which are involved in emotional processing and linked to the regulation of anxiety and depression [21,111]. In addition, compared to previous work, with the same herbal material but different forms of extract, essential oils delivered intranasally required a very small dosage but exerted their effects within a shorter time course [102]. Given these advantages, the use of essential oils for antidepressant development and its intranasal delivery are promising strategies and should be the focus of future research.

This study has some limitations that could be addressed in future research. First, the safety assessment of essential oils in mice administered intranasally was overlooked. Nasal delivery has been reported to be associated with irritation and dryness of the nasal mucosa, resulting in cracking, inflammation, and nosebleeds [112,113]. Hence, toxicity evaluation for the nasal mucosa and olfactory tissues is necessary before intranasal administration. The second limitation of this study was the tested dose (25 mg/kg), which was administered to mice for all 20 essential oils. Although we chose the dose based on previous studies [114,115,116,117], it should be noted that each essential oil has its own properties and characteristics; therefore, the effective doses and pathways in which they are absorbed or distributed are different. However, finding the right dose for each essential oil, including those that had never previously been studied or published, was relatively unfeasible. Thirdly, the compounds described in the molecular docking experiment were derived from a literature search, not a GC-MS analysis from our own samples. GC-MS analysis would be more appropriate for future in-depth research on each essential oil suggested in this paper. Finally, the resolution of the protein structure determines the accuracy of docking, yet there are currently no high-resolution data on other AMPAR subunits; hence, molecular docking could only be performed for the GluA2 subunit.

## 5. Conclusions

*Chrysanthemum morifolium* Ramat., *Zingiber officinale* Roscoe, *Santalum album* L., *Citrus reticulata* Blanco, *Atractylodes lancea* (Thunb.) DC., and *Myristica fragrans* Houtt. essential oils have been shown to possess neuroprotective or anti-neuroinflammatory effects in vitro and to exert rapid-acting antidepressant effects through intranasal delivery in mice. Notably, *Chrysanthemum morifolium* Ramat. and *Atractylodes lancea* (Thunb.) DC. essential oils and their volatile compounds, including atractylon, α-curcumene, α-farnesene, and selina-4(14),7(11)-dien-8-one, may be useful candidates for development as rapid-acting intranasal antidepressants through interactions with NMDARs and AMPARs. It is important for future work to validate the safety of these essential oils and compounds for intranasal delivery and the exact mechanism underlying their rapid-acting effects against depression. Additionally, they should be studied using a specific animal disease model.

## Figures and Tables

**Table 1:** Main compounds of essential oils.

No.	Essential Oil	Main Compounds					Ref.
1	*Magnolia biondii* Pamp.	Camphor	*trans*-Caryophyllene	1,8-Cineole	α-Pinene	β-Pinene	[44,45,46]
2	*Chrysanthemum* *morifolium* Ramat.	α-Curcumene	α-Farnesene	*n*-Heptadecane	Linoleic Acid	Nonadecane	[47,48]
3	*Zingiber* *officinale* Roscoe	Camphene	1,8-Cineole	β-Myrcene	β-Phellandrene	α-Pinene	[49,50,51]
4	*Santalum album* L.	(*Z*)-*trans*-α-Bergamotol	(*Z*)-Lanceol	(*E*)-Nuciferol	(*Z*)-α-Santalol	(*E*)-β-Santalol	[52,53,54]
5	*Citrus reticulata* Blanco	Limonene	Linalool	β-Myrcene	α-Pinene	γ-Terpinene	[55,56,57,58]
6	*Atractylodes lancea* (Thunb.) DC.	Atractylodin	Atractylon	β-Eudesmol	Elemol	Selina-4(14),7(11)-dien-8-one	[59,60,61]
7	*Myristica* *fragrans* Houtt.	Limonene	β-Myrcene	α-Phellandrene	α-Pinene	β-Pinene	[62,63,64]

**Table 2:** List of ligands.

No.	Compound	Pubchem CID
	*Reference compounds*	
1	Ketamine	3821
2	Ifenprodil	3689
3	TAK-653	56655833
	*Herbal compounds*	
1	Atractylodin	5321047
2	Atractylon	3080635
3	(*Z*)-*trans*-α-Bergamotol	5368743
4	Camphene	6616
5	Camphor	2537
6	*trans-*Caryophyllene	5281515
7	1,8-Cineole	2758
8	α-Curcumene	92139
9	Elemol	92138
10	β-Eudesmol	91457
11	α-Farnesene	5281516
12	*n*-Heptadecane	12398
13	(*Z*)-Lanceol	15560069
14	Limonene	22311
15	Linalool	6549
16	Linoleic Acid	5280450
17	β-Myrcene	31253
18	Nonadecane	12401
19	(*E*)-Nuciferol	6429177
20	α-Phellandrene	7460
21	β-Phellandrene	11142
22	α-Pinene	6654
23	β-Pinene	14896
24	(*Z*)-α-Santalol	11085337
25	(*E*)-β-Santalol	11031396
26	Selina-4(14),7(11)-dien-8-one	13986099
27	γ-Terpinene	7461

**Table 3:** Docking results of ligands towards GluN1, GluN2B, and GluA2 proteins.

No.	Ligand	Binding Energy (kcal/mol)		
		GluN1	GluN2B	GluA2
	Native ligand	−10.4	−10.7	−9.9
1	Ketamine	−8.2	−5.9	−4.9
2	Ifenprodil	−9.6	−9.7	−9.2
3	TAK-653	−8.6	−8.6	−9.2
4	Atractylodin	−7.2	−7.2	−6.0
5	Atractylon	−8.7	−8.8	−6.1
6	(*Z*)-*trans*-α-Bergamotol	−7.9	−7.0	−5.8
7	Camphene	−5.8	−5.3	−4.7
8	Camphor	−5.9	−4.6	−4.3
9	*trans-*Caryophyllene	−7.0	−6.4	−5.6
10	1,8-Cineole	−6.0	−5.6	−4.5
11	α-Curcumene	−8.8	−9.3	−7.0
12	Elemol	−7.2	−7.8	−6.4
13	β-Eudesmol	−7.0	−6.0	−6.2
14	α-Farnesene	−8.2	−8.4	−6.7
15	*n-*Heptadecane	−6.3	−6.6	−5.8
16	(*Z*)-Lanceol	−7.8	−8.1	−6.9
17	Limonene	−6.6	−7.5	−6.0
18	Linalool	−6.4	−6.7	−5.7
19	Linoleic Acid	−7.6	−7.2	−6.6
20	β-Myrcene	−6.4	−6.9	−5.0
21	Nonadecane	−6.8	−8.1	−6.9
22	(*E*)-Nuciferol	−8.1	−8.4	−6.9
23	α-Phellandrene	−6.7	−7.4	−6.1
24	β-Phellandrene	−6.4	−7.5	−6.1
25	α-Pinene	−6.1	−5.7	−4.6
26	β-Pinene	−6.1	−6.2	−4.4
27	(*Z*)-α-Santalol	−7.4	−6.4	−5.7
28	(*E*)-β-Santalol	−7.2	−5.5	−6.0
29	Selina-4(14),7(11)-dien-8-one	−8.8	−6.8	−7.6
30	γ-Terpinene	−7.0	−7.4	−5.9

The colors indicate binding energy values corresponding to binding affinity of ligands to the receptor subunits. Redder color: lower energy value, higher binding affinity; Whiter color: higher energy value, lower binding affinity.

**Table 4:** Summary of key research findings: neuroprotective, anti-neuroinflammatory effects in vitro and rapid-acting anti-anxiety and anti-depressant effects in vivo of 20 essential oils.

No.	Herb	In Vitro				In Vivo		
		Neuroprotective Dose (µg/mL)	Anti-Neuroinflammatory Dose (µg/mL)		Rapid Anti-Depressant Dose (mg/kg)		Rapid Anti-Anxiety Dose (mg/kg)	
			TNF-α	IL-6				
1	*Thuja orientalis* L.	1	-	-	-		-	
2	*Acorus gramineus* Sol.	-	-	-	-		-	
3	*Foeniculum vulgare* Mill.	0.1, 1	1	-	-		-	
4	*Magnolia biondii* Pamp.	1	-	-	-		-	
5	*Ligusticum striatum* DC.	1	1	1	-		-	
6	*Prunella vulgaris* L.	1	1	-	-		-	
7	*Chrysanthemum morifolium* Ramat.	0.1, 1	0.1, 1	-	25		25	
8	*Cinnamomum cassia* (Nees & T.Nees) J.Presl	1	0.1	0.1, 1	-		-	
9	*Zingiber officinale* Roscoe	1	-	-	25		-	
10	*Santalum album* L.	1	0.1, 1	1	25		-	
11	*Nardostachys jatamansi* (D. Don) DC.	1	-	-	-		-	
12	*Angelica acutiloba* (Siebold & Zucc.) Kitag.	0.1, 1	-	-	-		-	
13	*Aucklandia lappa* DC.	0.1, 1	-	1	-		-	
14	*Mentha arvensis* L.	1	-	1	-		-	
15	*Perilla frutescens* (L.) Britton	1	-	0.1, 1	-		-	
16	*Syzygium aromaticum* (L.) Merr. & L.M.Perry	0.1, 1	-	0.1, 1	-		-	
17	*Citrus reticulata* Blanco	0.1, 1	-	0.1, 1	25		-	
18	*Atractylodes lancea* (Thunb.) DC.	0.1, 1	-	0.1, 1	25		-	
19	*Agastache rugosa* (Fisch. & C.A.Mey.) Kuntze.	1	-	-	-		-	
20	*Myristica fragrans* Houtt.	0.1, 1	-	0.1, 1	25		25	

## Data Availability

The data presented in this study are available in this article.
